# Cardiac Kinetic Energy and Viscous Dissipation Rate From Radial Flow Data

**DOI:** 10.3389/fphys.2021.725104

**Published:** 2021-09-22

**Authors:** Sarah Frank, Junsung Lee, Jonas Lantz, Tino Ebbers, Shawn C. Shadden

**Affiliations:** ^1^Mechanical Engineering, University of California, Berkeley, Berkeley, CA, United States; ^2^Department of Health, Medicine and Caring Sciences, Linköping University, Linköping, Sweden

**Keywords:** blood flow, hemodynamics, ultrasonography, color Doppler echocardiography, kinetic energy, viscous dissipation rate

## Abstract

Recent studies have correlated kinetic energy (KE) and viscous dissipation rate (VDR) in the left ventricle (LV) with heart health. These studies have relied on 4D-flow imaging or computational fluid dynamics modeling, which are able to measure, or compute, all 3 components (3C) of the blood flow velocity in 3 dimensional (3D) space. This richness of data is difficult to acquire clinically. Alternatively, color Doppler echocardiography (CDE) is more widespread clinically, but only measures a single radial component of velocity and typically only over a planar section. Because of this limitation, prior CDE-based studies have first reconstructed a second component of velocity in the measurement plane prior to evaluating VDR or KE. Herein, we propose 1C-based surrogates of KE and VDR that can be derived directly from the radial component of the flow velocity in the LV. Our results demonstrate that the proposed 1C-based surrogates of KE and VDR are generally as well-correlated with the true KE and VDR values as surrogates that use reconstructed 2C flow data. Moreover, the correlation of these 1C-based surrogates with the true values indicate that CDE (3D in particular) may be useful in evaluating these metrics in practice.

## 1. Introduction

Kinetic energy (KE) and viscous dissipation rate (VDR) are two markers that have recently received attention for evaluation of cardiac function. In myocardial infarction patients, KE in the left ventricle (LV) has been shown to be significantly different than in controls, and also significantly different between myocardial infarction patients with and without thrombus formation in the LV (Garg et al., 2018, 2019). In other studies, significance has been found in KE measures taken at specific times in the cardiac cycle or when normalized in various ways. Systolic KE is higher in heart failure patients than healthy controls, and three distinct patterns in KE over time have been identified (Kanski et al., 2015). Peak diastolic KE, when indexed to stroke volume, is significantly lower in patients with Fontan circulation than healthy controls (Sjöberg et al., 2017). Peak diastolic KE, indexed to ventricular volume, decreases with age and peak diastolic KE of patients with left ventricular dysfunction is comparable to the older healthy individuals (Wong et al., 2016). KE has also been shown to be higher in tetralogy of Fallot patients than healthy controls, although these differences were not statistically significant (Jeong et al., 2015). In addition to KE spatially-averaged over the entire LV, KE in subsets of the ventricle has also been studied. For example, in a comparison of the KE measured throughout the ventricle vs. in only the short-axis base-to-apex plane, it was found that the proportion of KE captured in the short-axis plane was the same between controls and myocardial infarction without thrombus, but was higher in myocardial infarction with thrombus (Garg et al., 2019). These studies broadly indicate that KE is correlated with several different cardiac pathologies and may be useful for evaluation of cardiac diseases.

VDR, also referred to as viscous energy loss or viscous dissipation, has also been studied in intracardiac flow data. Studies have shown that in healthy subjects, VDR is lower than in diseased patients (Cibis et al., 2015; Kamphuis et al., 2018). Studies have also explored the relationship between vortex formation in the LV and patterns of VDR. It was found that viscous energy losses are correlated with different types of vortices in the LV, and viscous energy losses were higher in atrioventricular septal patients than healthy volunteers (Elbaz et al., 2017). In other studies, changes in the vortex in the LV associated with aortic regurgitation were correlated with higher viscous energy dissipation (Di Labbio and Kadem, 2018). In Fontan patients, energy losses were significantly elevated when compared to healthy controls (Kamphuis et al., 2019). And the natural angle of the mitral valve has been shown to minimize flow energy losses in the LV compared to other angles (Pedrizzetti and Domenichini, 2005). These studies indicate that VDR, like KE, is also correlated with heart health.

Most studies examining KE and VDR in the LV, including those listed above, have used data from computational fluid dynamic (CFD) simulations or 4D-flow magnetic resonance imaging (MRI). CFD and 4D-flow MRI can provide time-resolved measurements of all 3 components (3C) of the velocity over the 3-dimensional (3D) left ventricular volume. However, these methods are not routinely available. Therefore, while the richness of the flow data provided by these methods is ideal for evaluating KE and VDR, the clinical utility of this approach remains limited. Alternatively, color-Doppler echocardiography (CDE) is widely-available. However, a main limitation of CDE is that it only measures a single (beam-aligned) component of blood velocity (1C), and typically over a single planar view (2D). Therefore, CDE is most frequently used to assess transvalvular flow, where the flow field is mostly unidirectional, as opposed to intraventricular flow, which is more complex and three-dimensional. More recently, though, researchers have utilized CDE to evaluate intraventricular flow patterns by employing velocity reconstruction methods that aim to derive at least bidirectional (reconstructed 2C) flow information from CDE prior to, e.g., computing KE and VDR.

For example, a vector flow mapping method was used to reconstruct bi-directional, planar flow information from CDE, and VDR was examined as a potential indicator of flow quality (Itatani et al., 2013). This method was later used to determine baseline values of VDR in children (Hayashi et al., 2015), and to determine baseline values of VDR in adults (Akiyama et al., 2017). Similarly, in patients who had mitral valve surgery, ejection fraction and type of surgery were shown to affect postoperative VDR derived from CDE in the LV (Yoshida et al., 2018). In a study on diabetic patients, diastolic VDR computed from CDE was increased in diabetic patients compared to healthy controls and systolic VDR was increased in diabetic patients with uncontrolled blood glucose (Li et al., 2017). VDR has also been evaluated in dogs using CDE, where VDR increased when aortic regurgitation was present, and the diastolic VDR was shown to be proportional to the severity of regurgitation (Stugaard et al., 2015).

Bi-directional flow reconstruction from CDE is usually based on a planar flow approximation. While studies have shown that the planar flow approximation enables adequate reconstruction of the in-plane velocity field (Garcia et al., 2010) and calculation of diagnostic measures (Hendabadi et al., 2013; Bermejo et al., 2015; Rossini et al., 2017), others have shown that this assumption can be unreliable in some circumstances (Jang et al., 2015). Regardless, given the interest in intraventricular KE and VDR quantification in heart disease, it is interesting to evaluate if surrogates of such quantities can be effectively derived from CDE, either indirectly through reconstruction of 2D flow fields, or directly from a single component of velocity as provided from CDE. Namely, determining the correlation of KE and VDR measures directly derivable from CDE with true KE and VDR values can help establish the effectiveness of CDE for intracardiac hemodynamics evaluations that have traditionally relied on access to 4D flow MRI or CFD modeling.

In this paper, we propose and evaluate 1C-based surrogates of KE and VDR that are measured from a single component of velocity, analogous to the velocity measured from CDE. The proposed 1C surrogates, and reconstructed 2C surrogates introduced previously, are compared to “gold-standard” values obtained from 3D/3C intracardiac flow data. The proposed surrogates are evaluated on a single plane, representative of traditional CDE acquisition, and on multiple, parallel planes, to represent more recent/incipient techniques in 3D ultrasonography. In all cases however, we aim to utilize single (beam-aligned) velocity data to establish the effectiveness of CDE for intraventricular flow quantification.

## 2. Methods

### 2.1. Kinetic Energy

KE per unit volume in a fluid is defined as


$$ke(x,t)=\frac{1}{2}\rho \|u(x,t){\|}^{2}$$

where **u** is the fluid velocity, and ρ is the fluid density herein assumed to be constant. To be consistent with prior studies, we use a cylindrical coordinate representation. With such a representation, the total kinetic energy is given by


(1) $$KE_{3C}(t)=\frac{1}{2}\rho \int_{V}\left(u_{r}^{2}+u_{\theta }^{2}+u_{z}^{2}\right)rd\theta drdz$$

where *V* is assumed herein to be the intraventricular volume of the LV. This definition applies to 3D/3C velocity data. We define total kinetic energy over a single measurement plane (assumed to be a plane of constant *z*) from either 2C or 1C velocity information, respectively, as


(2) $$KE_{2C}(t;z_{i})=\frac{1}{2}\rho \int_{A}\left(u_{r}^{2}+u_{\theta }^{2}\right)rd\theta dr$$

(3) $$KE_{1C}(t;z_{i})=\frac{1}{2}\rho \int_{A}\left(u_{r}^{2}\right)rd\theta dr$$

where *z* = *z*_*i*_ defines the measurement plane, and *A* is the cross-sectional area of the intraventricular volume in the given measurement plane. The first definition is applicable to planar CDE data where the cross-beam velocity component *u*_θ_ is typically reconstructed from the measured velocity *u*_*r*_ and a planar flow assumption, and the second definition is applicable to standard planar CDE data where only the beam-wise velocity component *u*_*r*_ is available. Note, Equations (2) and (3) represent, respectively, the KE contributions from only 2 and 1 velocity component(s) and are integrated over an area and not volume. Thus, we refer to these as surrogates for, instead of estimates of, the complete KE as defined in Equation (1).

We also consider the above reduced order surrogates where Equations (2) and (3) are calculated on multiple, parallel planes, as might be possible with 3D ultrasonography methods. The result is a sum of the measures for the single planes:


(4) $$KE_{3D/2C}(t)=\sum_{i=1}^{n}KE_{2D,i}\Delta z$$

(5) $$KE_{3D/1C}(t)=\sum_{i=1}^{n}KE_{1D,i}\Delta z$$

where *n* is the number of planes and Δ*z* is the distance between the planes.

### 2.2. Viscous Dissipation Rate

VDR per unit volume (also referred to more simply as *viscous dissipation*) in a fluid is defined as


$$\begin{array}{l} \phi_{3D}(x,t)=\tau :\nabla u\\ \;=2\mu D:\nabla u\;(\text{incompressible},\text{Newtonian fluid}) \end{array}$$

where **τ** is the viscous stress tensor, **D** is the rate of strain tensor (i.e., the symmetric part of the velocity gradient, ∇**u**), and μ is the dynamic viscosity of the fluid. In cylindrical coordinates the total VDR is


(6) $$\begin{array}{l} \Phi_{3C}(t)=\int_{V}\mu (2\left[{\left(\frac{\partial u_{r}}{\partial r}\right)}^{2}+{\left(\frac{1}{r}\frac{\partial u_{\theta }}{\partial \theta }+\frac{u_{r}}{r}\right)}^{2}+{\left(\frac{\partial u_{z}}{\partial z}\right)}^{2}\right]\\ \;+{\left[r\frac{\partial }{\partial r}\left(\frac{u_{\theta }}{r}\right)+\frac{1}{r}\frac{\partial u_{r}}{\partial \theta }\right]}^{2}+{\left[\frac{1}{r}\frac{\partial u_{z}}{\partial \theta }+\frac{\partial u_{\theta }}{\partial z}\right]}^{2}\\ \;\;+{\left[\frac{\partial u_{r}}{\partial z}+\frac{\partial u_{z}}{\partial r}\right]}^{2})rd\theta drdz \end{array}$$

where *V* is again the intraventricular volume. This definition can only apply to 3D/3C velocity data. A corresponding 2C surrogate of the above VDR is proposed here by eliminating terms with *u*_*z*_ and with derivatives with respect to *z*. This results in a 2C VDR defined as


(7) $$\begin{array}{l} \Phi_{2C}(t;z_{i})=\mu \int_{A}(2\left[{\left(\frac{\partial u_{r}}{\partial r}\right)}^{2}+{\left(\frac{1}{r}\frac{\partial u_{\theta }}{\partial \theta }+\frac{u_{r}}{r}\right)}^{2}\right]\\ \text{ +}{\left[r\frac{\partial }{\partial r}\left(\frac{u_{\theta }}{r}\right)+\frac{1}{r}\frac{\partial u_{r}}{\partial \theta }\right]}^{2})rd\theta dr. \end{array}$$

where *z* = *z*_*i*_ defines the measurement plane and *A* is the LV chamber cross-sectional area in this plane. Similarly, for standard CDE data, only *u*_*r*_ is available and usually on a single plane. In such case a 1C VDR surrogate is derived from above by further neglecting *u*_θ_ to give


(8) $$\begin{array}{l} \Phi_{1C}(t;z_{i})=\mu \int_{A}\left(2{\left(\frac{\partial u_{r}}{\partial r}\right)}^{2}+2{\left(\frac{u_{r}}{r}\right)}^{2}+{\left(\frac{1}{r}\frac{\partial u_{r}}{\partial \theta }\right)}^{2}\right)\;rd\theta dr. \end{array}$$

Alternatively, a modified 1C surrogate can be derived by employing a planar-flow assumption. Namely, assume that the through-plane divergence is negligible on the imaging plane and thus the following 2D continuity equation holds


(9) $$0=\frac{1}{r}\frac{\partial u_{\theta }}{\partial \theta }+\frac{u_{r}}{r}+\frac{\partial u_{r}}{\partial r}\;.$$

Plugging Equation (9) into Equation (8) yields a 1D “planar-flow” surrogate of viscous dissipation rate as


(10) $$\begin{array}{l} \Phi_{pf1C}(t;z_{i})=\mu \int_{A}\left(4{\left(\frac{\partial u_{r}}{\partial r}\right)}^{2}+{\left(\frac{1}{r}\frac{\partial u_{r}}{\partial \theta }\right)}^{2}\right)\;rd\theta dr. \end{array}$$

We note here the role that the cylindrical coordinate system plays in the calculation of reduced order VDR surrogates. For example, in the definition of Φ_1*C*_ given in Equation (8), the $\frac{u_{r}}{r}$ term can be thought of as a “correction term” arising from the non-solenoidal nature of the unit coordinate vectors. In Cartesian coordinates, such terms do not arise and hence do not complicate dimensional reduction. However, ultrasound generally measures the radial component of the velocity field and divergence of the radial unit vector is non-zero, which yields this term. Notice, when assuming planar flow, the subsequently defined pf1C surrogate of VDR in Equation (10) leads to a more consistent way to reduce down to a 1D VDR measure in which this “correction term” becomes naturally eliminated.

Similar to KE, multi-plane surrogates of VDR were also considered, defined as follows:


(11) $$\Phi_{3D/2C}(t)=\sum_{i=1}^{n}\Phi_{2C,i}\Delta z$$

(12) $$\Phi_{3D/1C}(t)=\sum_{i=1}^{n}\Phi_{1C,i}\Delta z$$

(13) $$\Phi_{3D/pf1C}(t)=\sum_{i=1}^{n}\Phi_{pf1C,i}\Delta z$$

We note that for the multi-plane analyses, multiple parallel planes are used rather than multiple planes rotated along an axis as for a spherical coordinate system. Depending on the type of ultrasound system being used, a spherical coordinate representation may be appropriate. In such cases, the use of spherical coordinates would introduce additional “correction” terms in the reduced order viscous dissipation measures. However, including these terms herein would not be consistent with the 2D surrogates of viscous dissipation that are currently used in the literature.

### 2.3. Data Collection

Patient-specific intracardiac blood flow modeling from four patients was used to generate 3D/3C time-dependent velocity data inside the left ventricles to test the merit of 1C and 2C KE and VDR surrogates.

Coronary computer tomography (CT) data was acquired using a third-generation dual source CT (Siemens SOMATOM Force, Siemens Medical Solutions, Forcheim, Germany). The studies involving human participants were reviewed and approved by the regional ethical review board in Linköping, Sweden. The patients provided written informed consent to participate in this study. Retrospective image acquisition with electrocardiogram-triggered dose modulation was used to cover the whole cardiac cycle. A contrast dose of 335 mgI/kg (maximum weight 77 kg) was injected. A 4D reconstruction with a temporal resolution of 20 time frames per cardiac cycle and image resolution of 0.35×0.35 ×0.25 mm^3^ was used. From the 4D CT data, a map of the patient-specific wall motion was obtained, in order to prescribe the motion of the geometry in the flow model. In order to solve the flow equations, a volumetric mesh was created (Ansys ICEM 16.0, Ansys, Inc., Canonsburg, PA) based on the segmented wall. Due to the complex shape of the LV, a general tetrahedral mesh strategy was employed with a maximum allowed side length on the order of 0.75–1 mm.

**Figure 1:**
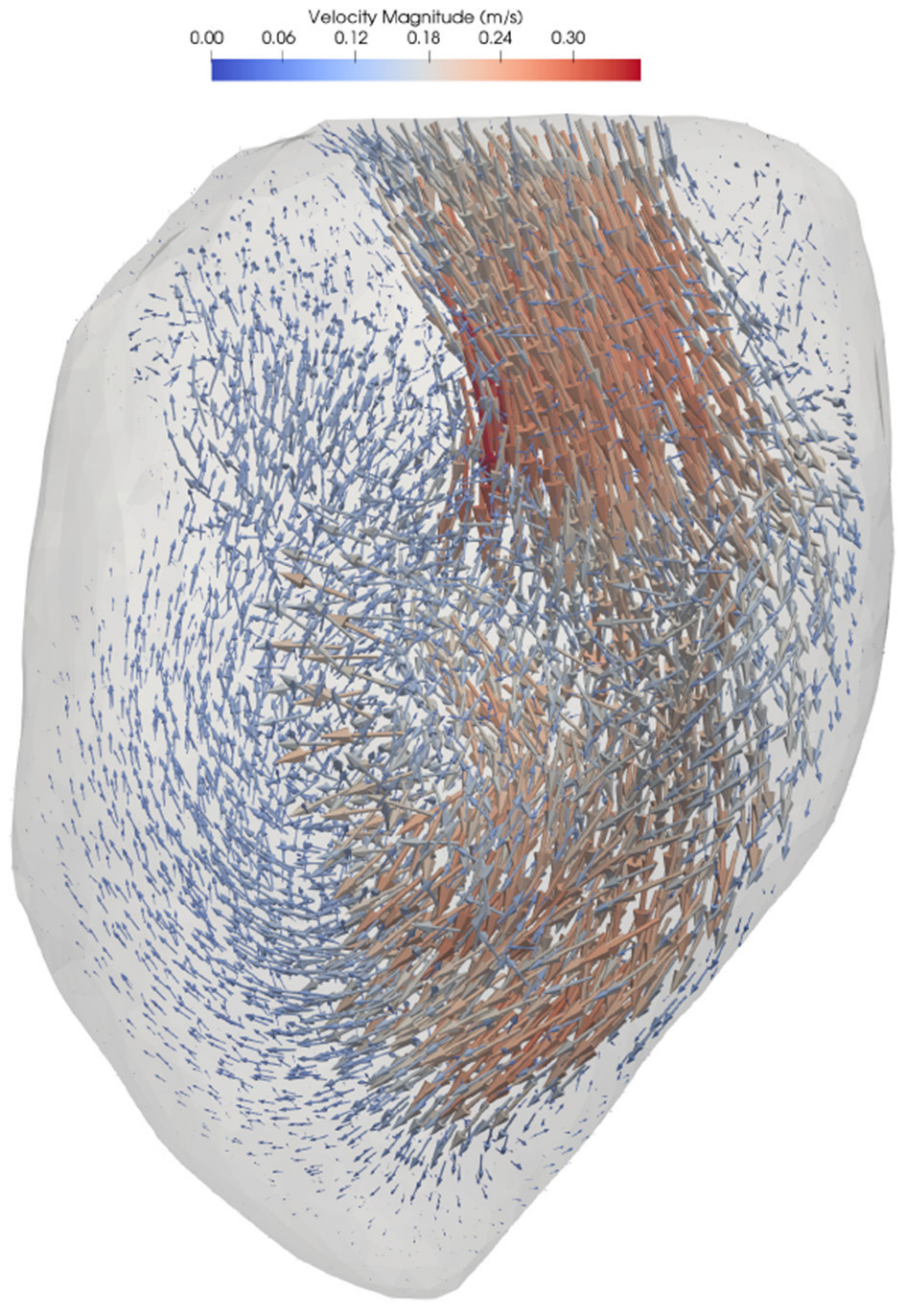
3D velocity field for representative LV.

Blood flow simulation was performed using Ansys CFX 16.0 (Ansys, Inc., Canonsburg, PA). The fluid was assumed to be incompressible with a density of 1,060 kg/m^3^ and dynamic viscosity of 3.5·10^−3^ Pa·s. The boundary conditions on the wall were specified as a displacement based on the image registration process. A zero relative static pressure was set at the four pulmonary veins, which resulted in approximately equal flow distribution into the atrium. Full details on the numerical methods can be found in Lantz et al. (2018). A representative flow field is shown in Figure 1.

Following blood flow simulation, the velocity data was projected and down-sampled to a structured Cartesian mesh in order to resemble a 4D-flow MRI measurement with a resolution of 2–3 mm voxels and approximately 20 frames per cycle (one had 19 frames and the rest had 20 frames per cycle). This down-sampled data was then used to extract virtual CDE data as described next.

Virtual CDE was obtained by projecting the velocity data to a virtual imaging plane (or planes in the cases of multi-plane analyses) and extracting only the radial component *u*_*r*_ of the velocity field. KE and VDR were then calculated directly from *u*_*r*_. In the case of VDR, Φ_1*C*_ was computed from Equation (8) and Φ_*pf*1*C*_ was computed from Equation (10). The 2C surrogates were calculated in two ways. First, idealized 2C surrogates were obtained using the radial and azimuthal components of the velocity directly from the virtual 4D-flow data. Alternatively, more realistic 2C surrogates were obtained using the virtual CDE *u*_*r*_ along with a reconstructed *u*_θ_ calculated using the method described in Garcia et al. (2010). The complete list of KE and VDR surrogates are in Table 1. All surrogates were calculated from flow data on a single nominally apical long-axis imaging plane (“single-plane analyses”), and from flow data on multiple parallel long-axis planes (“multi-plane analyses”). All surrogates were compared to the baseline 3D/3C KE and VDR from the virtual 4D-flow velocity field across the entire LV.

**Table 1:** Abbreviations for KE and VDR surrogates.

**Abbreviation**	**Definition**
2D/1C KE [VDR]	Computed from Equation (3) [Equation 8]
3D/1C KE [VDR]	Computed from Equation (5) [Equation 12]
2D/pf1C VDR	Computed from Equation (10) based on planar flow (pf) assumption
3D/pf1C VDR	Computed from Equation (13) based on planar flow (pf) assumption
2D/2Ct KE [VDR]	Computed from Equation (2) [Equation 7] using true values of *u*_θ_
2D/2Cr KE [VDR]	Computed from Equation (2) [Equation 7] using reconstructed values of *u*_θ_

#### 2.3.1. Variability of Probe Location and Orientation

**Figure 2:**
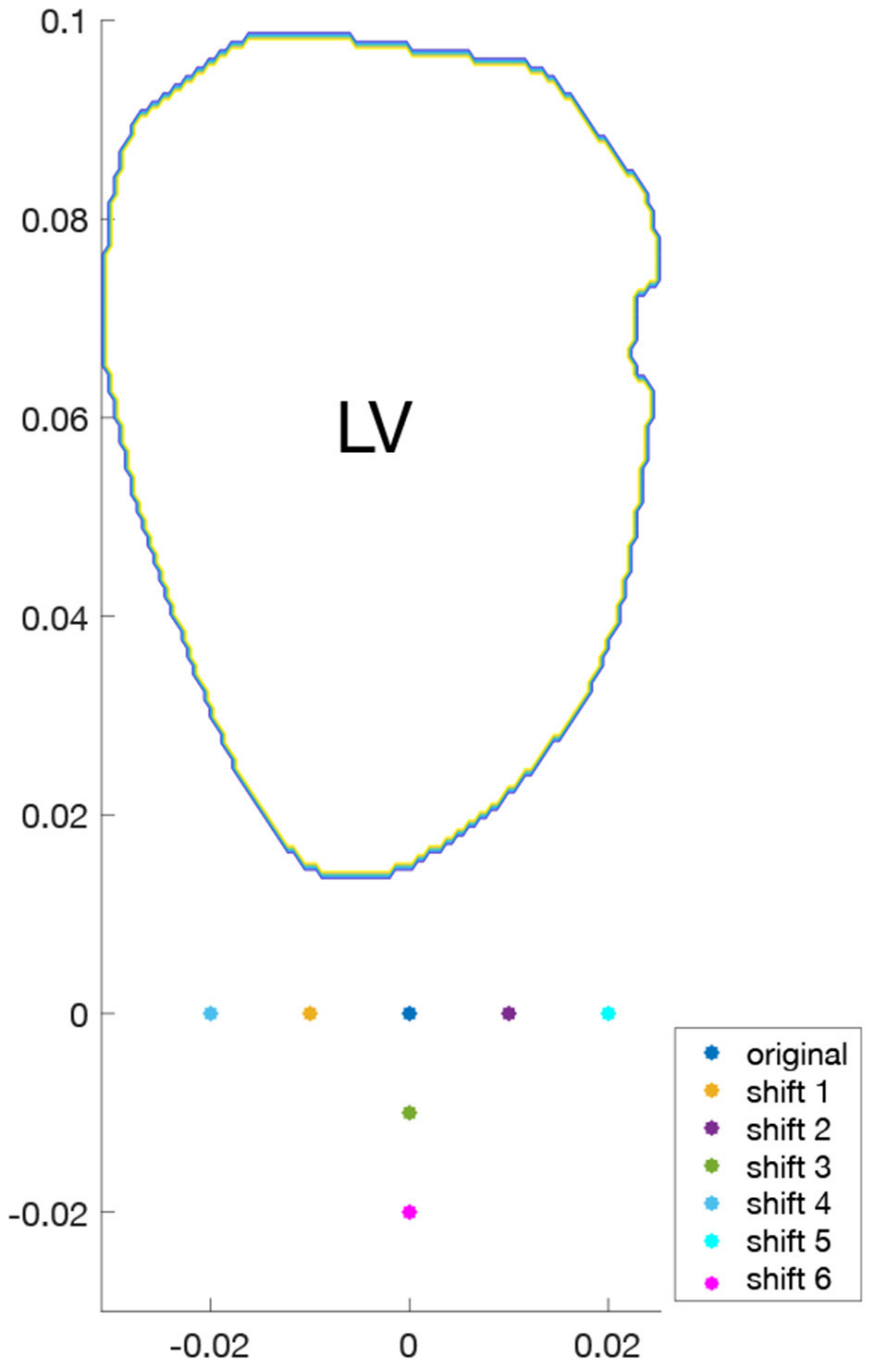
Shifts in probe locations for representative LV.

**Figure 3:**
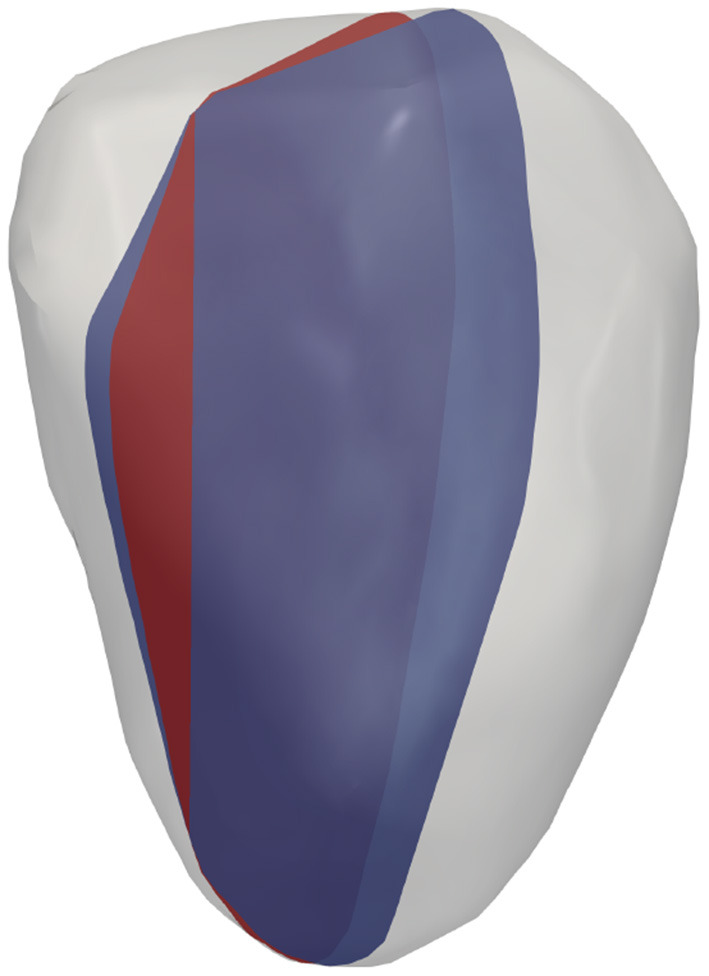
Virtual imaging planes for representative LV.

To study the effects of probe location on surrogates of KE and VDR, for each of the four hearts examined, different acquisitions were simulated by varying the probe location. For both single-plane and multi-plane analyses, separate acquisitions were simulated by shifting the probe location, as shown in Figure 2. Because the radial direction at any given coordinate depends on the probe location (origin), each probe location samples a different direction of the velocity vector as its radial component. In addition, for the single-plane analyses, the orientation of the measurement plane was perturbed, as shown in Figure 3, and the various probe locations were considered for both measurement plane orientations. Data from all different probe locations and orientations were combined for statistical analysis.

#### 2.3.2. Variability Due to Noise and Data Resolution

The effects of noise and resolution were also explored by modifying the original (virtual 4D-flow) data and recalculating all reduced order surrogates of KE and VDR. Specifically, a uniformly distributed multiplicative noise of ±25% was assumed, i.e.,


$${\tilde{u}}_{i}=u_{i}(1+\epsilon )$$

where ϵ is uniform random variable between −0.25 and 0.25. To study the effects of data resolution, the resolution was reduced by half in both in-plane directions, resulting in an effective sampling of only 25% of the original data. The introduction of noise and down sampling was applied for all probe locations and orientations considered.

### 2.4. Statistical Methods

True KE and VDR values were computed at the ≈20 time points during the cardiac cycle for the four patient models using the 3D/3C velocity information in Paraview (ver. 5.9.1). At each time point, the various KE and VDR surrogates listed in Table 1 were also computed. Specifically, each surrogate was computed based on each of the 7 probe locations, and for the single-plane analyses (sections 3.1, 3.3) this was done in 2 planes, resulting in 14 realizations of each surrogate at a given time point. These 14 realizations (or 7 in the multi-plane analyses) of each given KE (or VDR) surrogate were matched with the corresponding true KE (or VDR) value at that time point; these pairs were aggregated over all patient models and time points, and linear regression between all these pairs was performed. The coefficient of determination (*R*^2^) was then computed for the linear regression to measure the correlation between a given KE/VDR surrogate and the true KE/VDR value. Note, *R*^2^ also represents the square of the sample correlation coefficient.

## 3. Results

### 3.1. Kinetic Energy: Single-Plane Analyses

**Figure 4:**
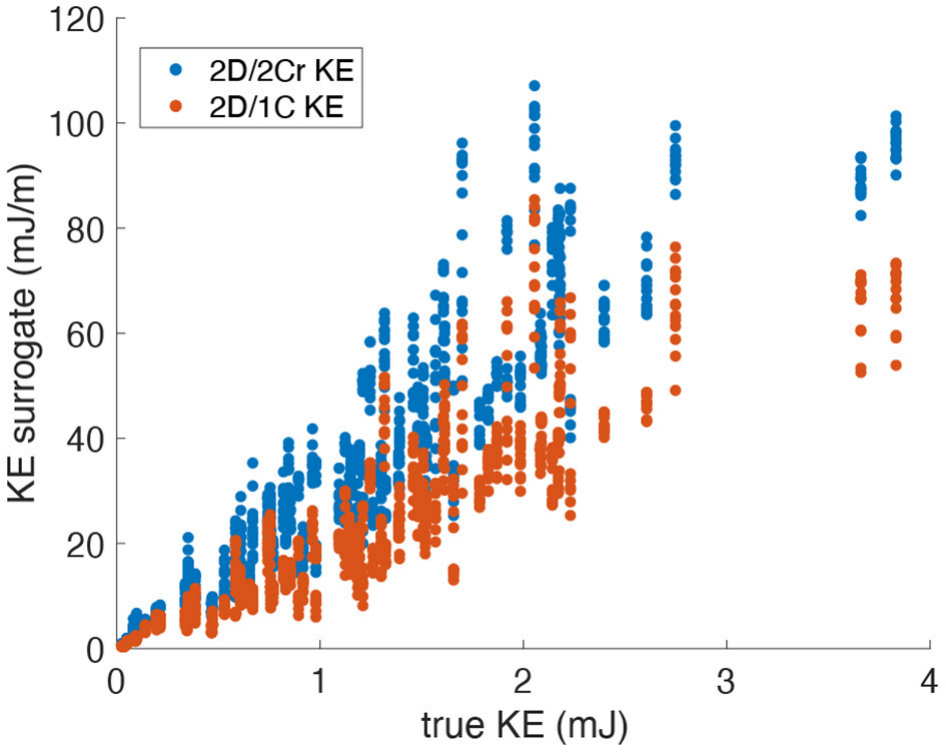
Scatter plot of KE surrogates measured from a single measurement plane vs. the true KE values. Data points span all patients, time points and probe locations/orientations (original grid, no noise).

Figure 4 plots the 2D/1C and 2D/2Cr surrogates of KE against the true 3D/3C KE values. The data points are collectively from all probe locations and measurement planes, for all four patient-specific LV models, and all time points over the cardiac cycle. We note that the specific values of 2D/1C, 2D/2Cr, and 3D/3C KE should not be directly compared since they are not the same quantities. In particular, one should not compare the slope of the 2D/2Cr surrogate against the slope of the 2D/1C surrogate. Instead, the correlation of each surrogate with the true 3D/3C value is more meaningful.

**Figure 5:**
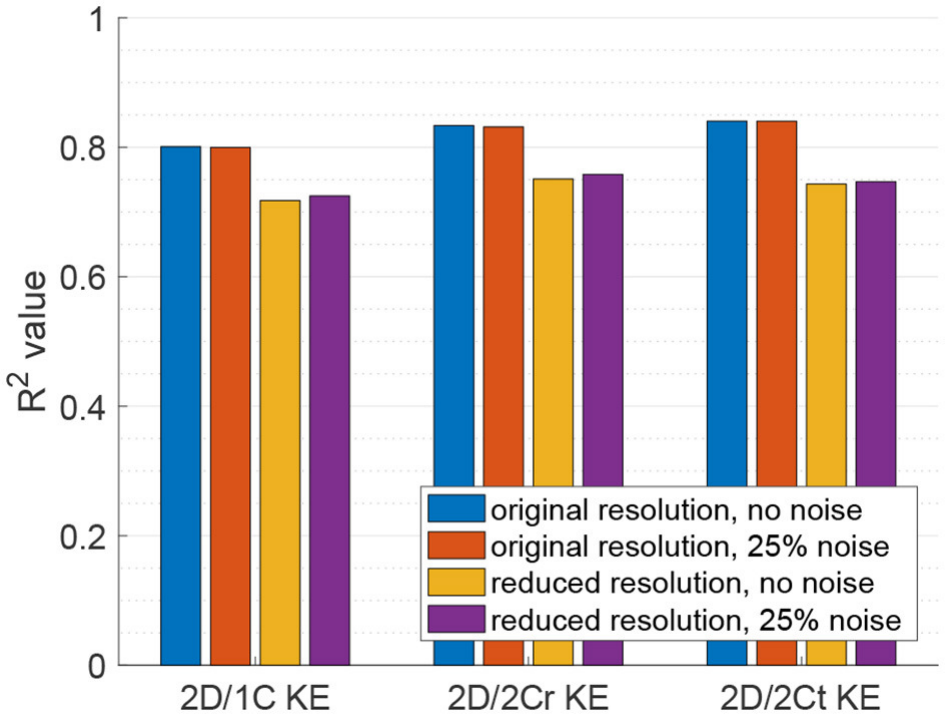
Coefficient of determination (*R*^2^) of KE surrogates measured from a single measurement plane to the true 3D/3C KE.

Figure 5 plots the coefficient of determination (*R*^2^), hence correlation, of all reduced order KE surrogates to the true 3D/3C KE values. Results using the original grid resolution and no added noise are shown in blue. The correlation of the (idealized) 2D/2Ct surrogate was around *R*^2^ = 0.84, which indicates that a KE evaluated from just 2 components of velocity in a single measurement plane correlates strongly with the full KE computed from all 3 components of velocity over the entire 3D left ventricular volume. However, CDE does not provide the azimuthal component of velocity, so the 2D/1C and 2D/2Cr surrogates are more realistic. Nonetheless, both the 2D/1C and 2D/2Cr KE surrogates maintained reasonably good correlations, *R*^2^ = 0.8 and *R*^2^ = 0.83, respectively, with the true 3D/3C KE values.

When noise was added to the data, the correlation of all reduced order KE surrogates to the true 3D/3C KE values remained relatively unchanged (Figure 5, red). When the resolution of the data was reduced, the *R*^2^ values between the true 3D KE and the reduced order surrogates dropped by roughly 10% in all cases as compared to the *R*^2^ values from the original resolution data (Figure 5, yellow). When both the resolution of the data was reduced and noise was added to the data (Figure 5, purple), the *R*^2^ values were nearly unchanged from those obtained with only reducing the resolution of the data. These results indicate that reduction in resolution is more important to KE than unbiased noise.

### 3.2. Kinetic Energy: Multi-Plane Analyses

**Figure 6:**
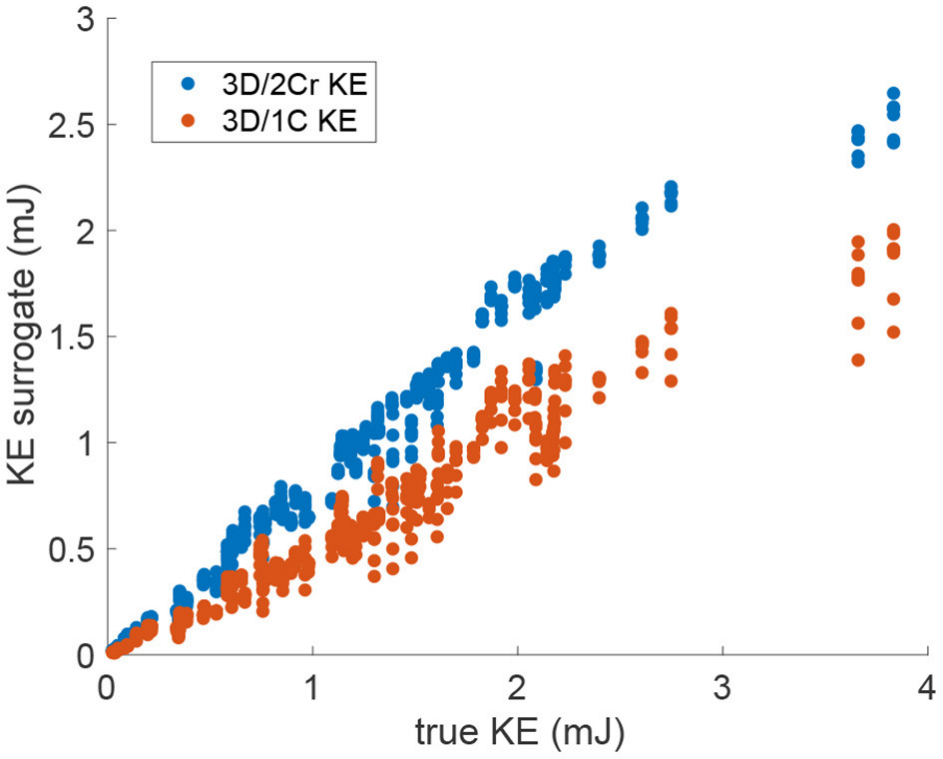
Scatter plot of the multi-plane 3D/2Cr KE and 3D/1C KE values versus the true 3D/3C KE values. Data points span all patients, time points and probe locations. (Original grid, no added noise).

**Figure 7:**
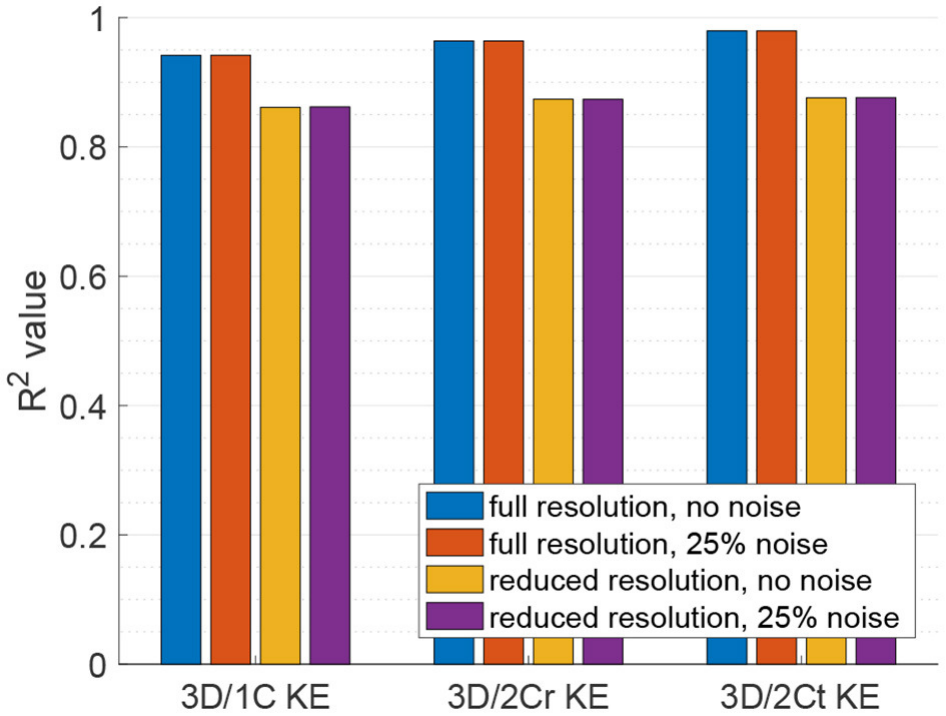
Coefficient of determination (*R*^2^) of the reduced order KE surrogates measured from multiple parallel planes to the true 3D/3C KE.

For KE measured from virtual multi-plane ultrasound, the reduced order surrogates were strongly correlated with true 3C values of KE (Figure 6). Indeed, as shown in Figure 7, the *R*^2^ values between the reduced order KE surrogates and the true 3D/3C KE values increased compared to the single-plane data, with all *R*^2^ values above 0.94 for the original grid (Figure 7, blue). Similarly to the single-plane data, reducing the grid resolution caused the *R*^2^ values to drop by about 10%, closer to 0.86 (Figure 7, yellow). Adding noise to the data did not have a significant effect on the correlations (Figure 7, red and purple).

### 3.3. Viscous Dissipation Rate: Single-Plane Analyses

**Figure 8:**
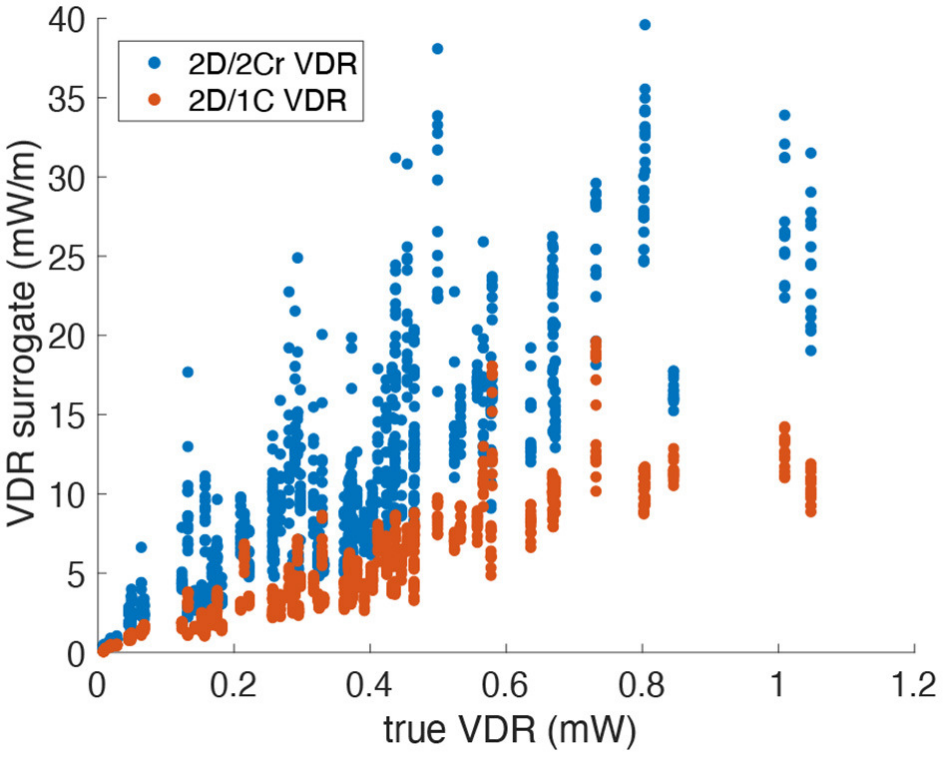
Scatter plot of VDR surrogates measured from a single measurement plane vs. the true VDR values. Data points span all patients, time points and probe locations/orientations (Original grid, no noise).

Figure 8 plots the 2D/1C and 2D/2Cr VDR values against the true 3D/3C VDR values. The data points are collectively from all probe locations/orientations, all patient models, and all time points in the cardiac cycle. VDR values from the different types of surrogates do not measure precisely the same quantity and thus absolute values (or slopes) should not be directly compared between these measures.

**Figure 9:**
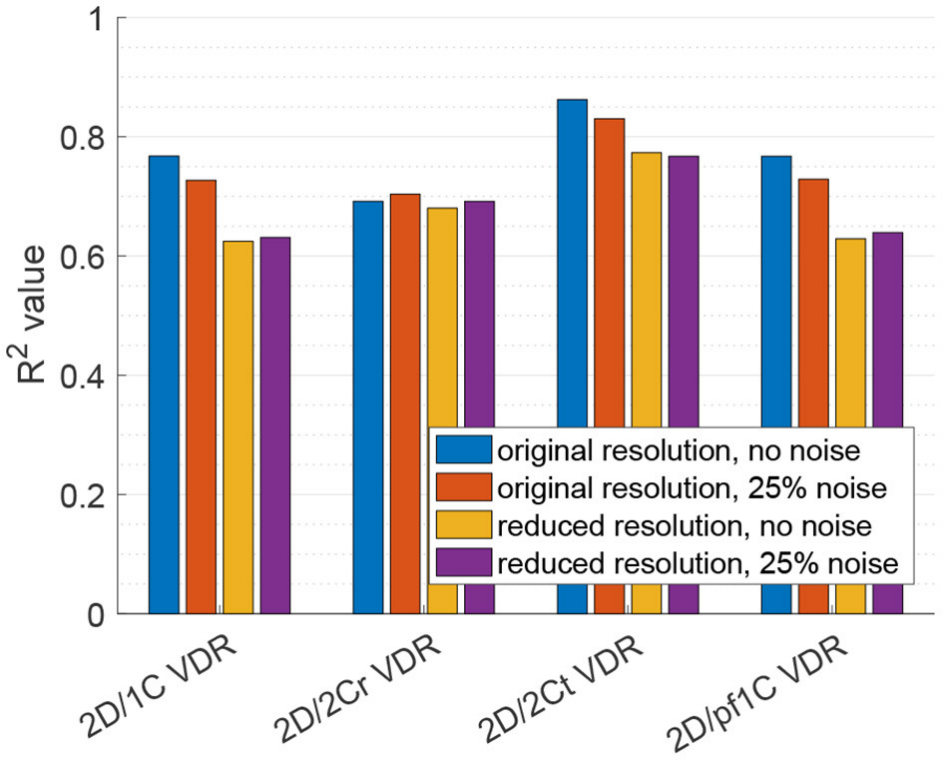
Coefficient of determination (*R*^2^) of VDR surrogates measured from a single measurement plane to the true 3D/3C VDR.

Figure 9 plots the correlation, *R*^2^, of each reduced order surrogate against the true 3D/3C VDR value. The idealized 2Ct VDR surrogate had the highest correlation with an *R*^2^ of approximately 0.86. This indicates that a 2D surrogate of VDR only from the long-axis apical plane is well-correlated with the true VDR computed from all 3 velocity components over the entire 3D LV volume. However, since CDE does not provide the azimuthal component of velocity, the 2Cr, 1C, and pf1C surrogates are more practical. Both the pf1C and 1C surrogates had *R*^2^ values of approximately 0.77, which were higher than the *R*^2^ value for the 2Cr surrogate, which was about 0.69. These results indicate that the reconstruction process may be detrimental to the calculation of VDR, which is also apparent from the increased scatter of the 2Cr values in Figure 8.

For the 2D/1C and 2D/pf1C VDR surrogates, addition of the noise to the velocity data lowered the *R*^2^ values by around 5%, and the reduction in data resolution lowered *R*^2^ values by around 18%. Reducing data resolution and adding noise together resulted in *R*^2^ values close to those observed from reduction of data resolution alone. For the 2D/2Cr surrogate based on reconstruction of the 2C flow field over the measurement plane, the correlation was already relatively low (*R*^2^ ≈ 0.69) and the addition of noise or reduction of data resolution did not change this correlation significantly, likely because it was already not strongly correlated.

### 3.4. Viscous Dissipation Rate: Multi-Plane Analyses

**Figure 10:**
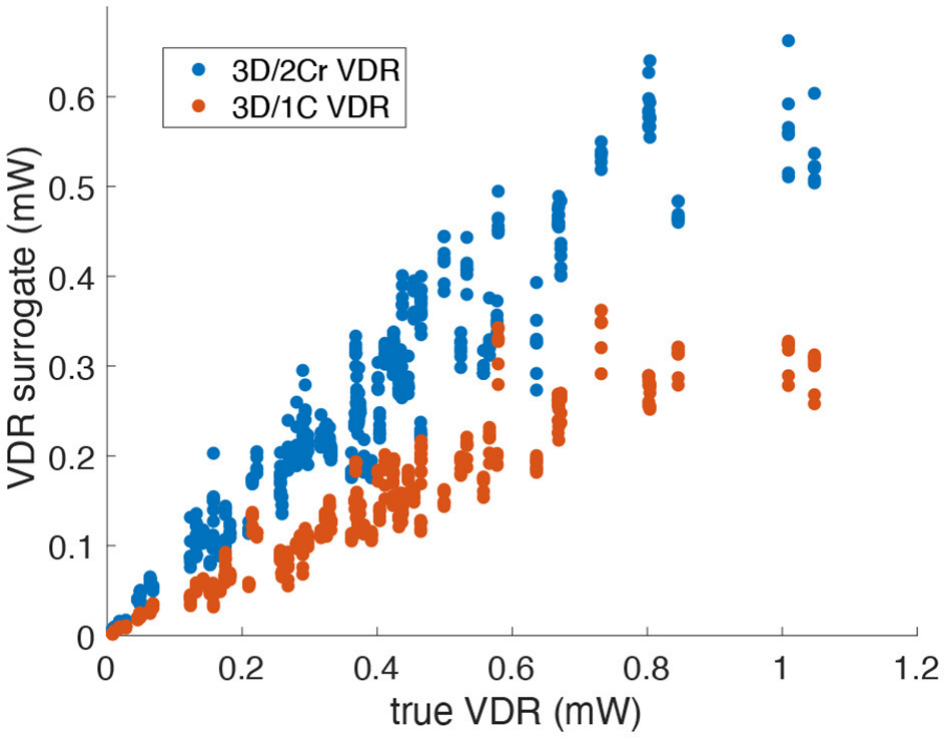
Comparison of multi-plane reduced-order surrogates of VDR to the true 3D measured VDR (original grid, no noise).

**Figure 11:**
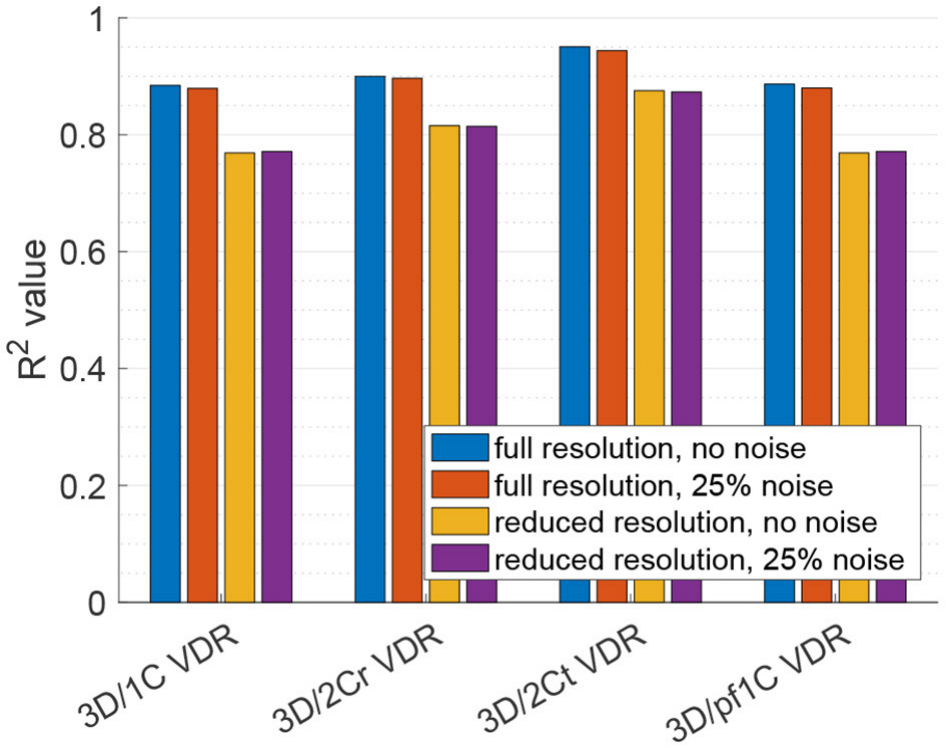
Coefficient of determination (*R*^2^) of VDR surrogates measured from a multi-plane measurement to the true VDR.

For VDR measured from multi-plane data, the *R*^2^ values between the reduced order surrogates and the true 3D/3C-based values increased compared to surrogates from a single plane. Figure 10 plots the 3D/1C and 3D/2Cr VDR values against the true VDR values, and Figure 11 plots the *R*^2^ values. All reduced order surrogates had an *R*^2^ value greater than 0.88, indicating that both 1C- and 2C-based surrogates of VDR are well-correlated to true VDR when multi-plane data is used. Unlike in the single-plane analysis, the reconstructed 2C VDR performed similarly to the 1C VDR surrogates. Similar to the single-plane analysis, the pf1C VDR and 1C VDR were similarly correlated to true VDR.

When noise was added to the full resolution data (Figure 11, red bars), the *R*^2^ values decreased slightly by around 1%. When the data resolution was reduced (Figure 11, yellow bars), the *R*^2^ values decreased by around 13% for the 1C-based VDR surrogates and around 10% for the 2C-based surrogates. The addition of noise to the reduced resolution data did not further change the correlations (Figure 11, purple bars).

## 4. Discussion

Reduced order surrogates of KE and VDR were calculated from LV flow data in a single plane and in multiple parallel planes. Using data from just a single plane, the correlation of KE and VDR computed from the 2 in-plane velocity components were well-correlated (*R*^2^ ≈ 0.85) to the true KE and VDR values from the entire 3D LV volume and using all 3 components of velocity. However, it is not routinely possible to measure bi-directional velocity information with CDE, and for this reason 2D/1C and 2D/2Cr surrogates were evaluated. For KE, the 2D/1C and 2D/2Cr surrogates were reasonably well-correlated with the true KE values for the entire LV, and were nearly as well-correlated as the 2D/2Ct KE surrogate. Thus, radial flow data on just a single measurement plane may be sufficient for assessing KE trends in the LV. For VDR, the 2D/1C surrogate was better correlated with the true 3D/3C VDR than the 2D/2Cr surrogate. This suggests that in order to acquire VDR information from single-plane ultrasonography, it may be more effective to calculate it directly from the radial flow information, rather than from a 2C surrogate that involves reconstructing the azimuthal component of the velocity.

KE surrogates were generally better correlated to true LV KE, than VDR surrogates were to true LV VDR. Therefore, assessment of KE from limited flow data appears more reliable than assessment of VDR from limited flow data. This is likely because KE can be computed directly from the velocity data, whereas VDR is based on velocity gradients that must be indirectly computed from the velocity measurements–a process that introduces additional error. Likewise, we also observed VDR was generally more affected by reduction in resolution or addition of noise to the velocity data.

The relationship between disease and KE or VDR has been explored in other studies, especially in 3D using MRI (Kanski et al., 2015; Wong et al., 2016; Sjöberg et al., 2017; Kamphuis et al., 2018; Garg et al., 2019), and in some studies in 2C using color-Doppler ultrasound (Stugaard et al., 2015; Yoshida et al., 2018). Although we observed that KE may be more effectively evaluated from limited flow measurements than VDR, we also observed that true 3D/3C KE and the true 3D/3C VDR were correlated (*R*^2^ ≈ 0.8); i.e., LVs with higher KE generally had higher VDR. Because of the no-slip condition on the endocardium (low near-wall flow velocities) and the confinement of the flow in the LV, when there are high velocity magnitudes (high KE), there will concomitantly be large spatial transitions between high and low velocity magnitudes (high VDR). Thus, correlation between KE and VDR may be expected. Nonetheless, KE and VDR should be further studied in tandem in the context of disease, to determine if they can provide compellingly unique information or if measuring just KE will provide sufficient evaluation.

In considering the effect of noise on surrogates for KE and VDR, it was found that reduced order surrogates of KE were more robust to noise than reduced order surrogates of VDR. Indeed, noise had negligible effect in the correlation of the reduced order KE surrogates to the true KE. This result may be expected since KE is derived directly from velocity magnitude, and unbiased noise will both increase and decrease velocity magnitudes and these changes could generally cancel out when integrated over the LV volume. On the other hand, VDR is based on velocity gradients and noise in the velocity field tends to increase gradients. Indeed, VDR surrogates generally became less correlated (≈7% reduction) to true VDR values when noise was added.

We found that calculations of VDR are sensitive to grid resolution, which confirms findings in Cibis et al. (2015). For the LV flow data on a single plane, we saw that on a coarse grid, the coefficients of determination between the reduced order surrogates and the 3D values of VDR decreased more than for KE. The influence of grid resolution is an important consideration when evaluating VDR because different machines/settings may results in different measurement resolutions, so it may be difficult to achieve consistent comparison of VDR values between hospitals/clinics or even longitudinally for a patient.

We evaluated variability of KE and VDR surrogates to changes in probe location and orientation, particularly for the 2D/1C surrogates since these were generally as well-correlated to the true values as the 2D/2Cr surrogates but were expected to be more sensitive to probe location and orientation than the 3D/1C surrogates. It was observed (see Supplementary Material) that shifting the probe outward (shifts 3 and 6) generally did not have much affect on 2D/1C KE values. When the probe was shifted closer to the intraventricular septum (shifts 2 and 5) the 2D/1C KE/VDR values were generally lower. Alternatively, when the probe was shifted toward the mitral valve (shifts 1 and 4), the 2D/1C KE/VDR values were generally higher. Despite these changes, the correlation of the 2D/1C surrogates to their true 3D/3C values was generally maintained over time irrespective of probe location and orientation.

Calculating reduced order surrogates on multiple planes of velocity data significantly increased the correlations between the reduced order surrogates and the true 3D/3C values. In particular, 1C and 2Cr KE surrogates were very strongly correlated (*R*^2^ > 0.94) to true values. The multi-plane surrogates were computed on multiple parallel planes. Depending on the ultrasonography system, measurement planes may not be entirely parallel. While it is unclear this would lead to significant difference, it compels further study into 3D ultrasonography systems, which could be useful in intracardiac flow quantification.

### 4.1. Limitations

This study is limited by the lower number of patient models. Being a feasibility study, it was primarily focused on the capability of intraventricular KE and VDR to be assessed from limited flow information. Indeed, it is not obvious that 1C flow information, particularly on a single measurement plane, could be of value in assessing highly 3D and complex flow. For this determination, we chose patient-specific CFD as the gold-standard, and then performed “virtual CDE” on that data, since comparing KE/VDR from 4D-flow measurements and CDE would introduce a host of inaccuracies that would confound this determination. Nonetheless, future studies, particularly those focused on applications of disease diagnosis, should consider a greater number of patients and true CDE measurements to better establish the clinical utility of CDE in evaluation of KE or VDR.

## 5. Conclusions

Reduced order measures of kinetic energy and viscous dissipation rate derived from only the radial component of the flow were shown to be correlated with true values of kinetic energy and viscous dissipation rate derived from all 3 components of the flow inside the left ventricle. These finding suggest that the direct assessment of kinetic energy and viscous dissipation rate from color Doppler echocardiography may be of clinical value in echo-based evaluation of cardiac function. Moreover, assessment of kinetic energy trends from radial flow data was more reliable than evaluation of viscous dissipation rate from radial flow data, particularly if the radial flow data was only available on a single measurement plane, had coarser resolution, or was noisy.

## Data Availability Statement

The datasets presented in this article are not readily available however the datasets can be made available for purposes and under conditions described in the ethical approval. Requests to access the datasets should be directed to Tino Ebbers, tino.ebbers@liu.se.

## Ethics Statement

The studies involving human participants were reviewed and approved by the regional ethical review board in Linkoping, Sweden. The patients provided written informed consent to participate in this study.

## Author Contributions

SF and SS conceptualized the study, interpreted results, and drafted the manuscript. SF and JLee developed the methods. SF, JLee, and SS performed computations. JLee, JLan, and TE interpreted results and revised the manuscript. All authors contributed to the article and approved the submitted version.

## Conflict of Interest

The authors declare that the research was conducted in the absence of any commercial or financial relationships that could be construed as a potential conflict of interest.

## Publisher's Note

All claims expressed in this article are solely those of the authors and do not necessarily represent those of their affiliated organizations, or those of the publisher, the editors and the reviewers. Any product that may be evaluated in this article, or claim that may be made by its manufacturer, is not guaranteed or endorsed by the publisher.
