# Supplementary figures and images for: Cardiac Kinetic Energy and Viscous Dissipation Rate From Radial Flow Data
Source: Front Physiol. 2021 Sep 22;12:725104. doi: 10.3389/fphys.2021.725104 (PMC8493223; doi:10.3389/fphys.2021.725104)

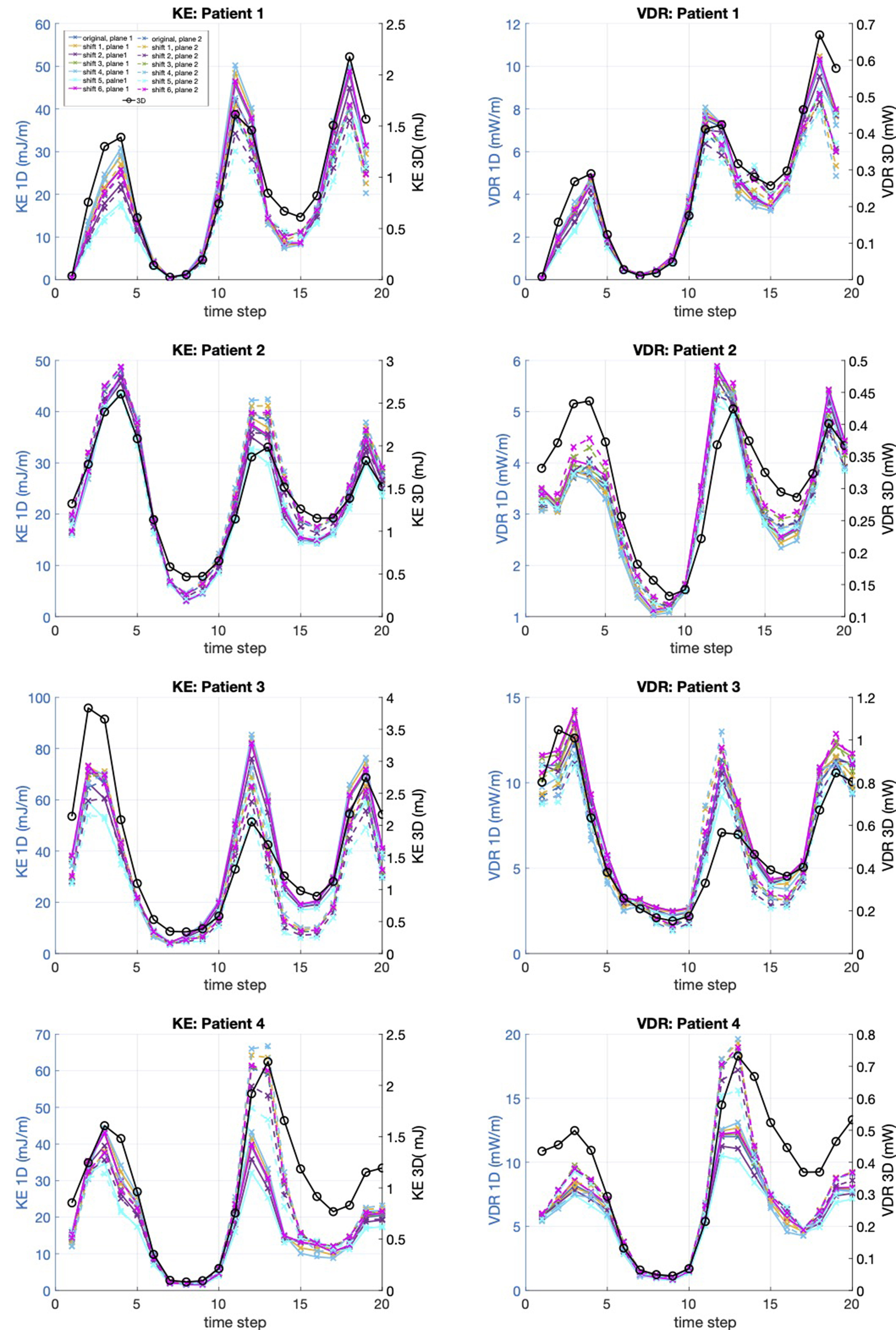

Supplement: Supplementary file 1 [file Image_1.JPEG]
